# Tumor Expression of CD83 Reduces Glioma Progression and Is Associated with Reduced Immunosuppression

**DOI:** 10.1158/2767-9764.CRC-24-0281

**Published:** 2024-12-30

**Authors:** Malcolm F. McDonald, Rachel Naomi Curry, Isabella O’Reilly, Brittney Lozzi, Alexis Cervantes, Zhung-Fu Lee, Anna Rosenbaum, Peihao He, Carrie Mohila, Arif O. Harmanci, Akdes Serin Harmanci, Benjamin Deneen, Ganesh Rao

**Affiliations:** 1 Medical Scientist Training Program, Baylor College of Medicine, Houston, Texas.; 2 Development, Disease Models, and Therapeutics, Baylor College of Medicine, Houston, Texas.; 3 Center for Cell and Gene Therapy, Baylor College of Medicine, Houston, Texas.; 4 Department of Pathology, Texas Children’s Hospital, Houston, Texas.; 5 School of Biomedical Informatics, University of Texas Health Science Center, Houston, Texas.; 6 Center for Cancer Neuroscience, Baylor College of Medicine, Houston, Texas.; 7 Department of Neurosurgery, Baylor College of Medicine, Houston, Texas.

## Abstract

Malignant glioma, the most lethal form of brain cancer, presents with an immunosuppressive microenvironment that obstructs tumor cell clearance and hampers immunotherapeutic interventions. Despite advancements in characterizing cellular and extracellular profiles in cancer, the immunosuppressive mechanisms specific to glioma remain poorly understood. We conducted single-cell RNA sequencing of glioma samples, which revealed a select subset of human and mouse glioma cells that express CD83, a marker associated with mature antigen-presenting cells. To investigate the impact of tumor cell CD83 expression on glioma outcomes, we used an immunocompetent mouse model of glioma, bioinformatic analyses of human samples, and *in vitro* assays. Our findings revealed that CD83^+^ tumor cells contribute to tumor growth suppression and are associated with enhanced cytotoxic T-cell profiles and activated CD8^+^ T cells. Increased proinflammatory cytokines were identified in CD83-overexpressing tumor conditions, which were also correlated with long-term CD8^+^ antitumor responses. Importantly, tumor-derived CD83 could mediate communication with T cells, altering the immune microenvironment to potentially enhance immune-related tumor clearance. Collectively, our data suggest that tumor cell expression of CD83 supports the endogenous antitumor T-cell constituency in malignant glioma. Future research endeavors may aim to further investigate whether CD83 expression can enhance immunotherapeutic approaches and improve patient outcomes.

**Significance:**

Immunosuppression in malignant glioma remains a barrier to therapeutic development. CD83 overexpression in human and mouse glioma increases survival. CD83^+^ tumor cells promote signatures related to cytotoxic T cells, enhanced activation of CD8^+^ T cells, and increased proinflammatory cytokines. These findings suggest that tumor-expressed CD83 could mediate tumor–immune communications.

## Introduction

Malignant gliomas are the most common and lethal form of central nervous system tumors and are resistant to immunotherapeutic interventions (1). This resistance is in part mediated by a robust immunosuppressive microenvironment comprised of tumor-associated macrophages (TAM), regulatory T cells, and glioma cell-derived immunomodulating factors (2–7). Increased exhausted and anergic T cells have been directly correlated with poorer survival, prompting the development of therapeutics that can overcome dysregulated T-lymphocyte function (8–11). Although T-cell exhaustion has been overcome in other cancers with the use of immune checkpoint inhibitors, these treatments have failed to improve survival for patients with glioma, highlighting the need for further insights into how these processes are dysregulated in glioma (12–17).

Cytotoxic CD8^+^ T lymphocytes (CTL) are frequently hyporesponsive in cancer (18). These immunosuppressive CTLs frequently manifest as anergic or exhausted, caused by incomplete activation or coinhibitory signaling in the former (19), or persistent antigen exposure in the latter (20). Anergic and exhausted CTLs are defined by the absence of effector functions, including the production of T cell–activating cytokines including IL-2, IFNγ, and TNFα, and enhanced expression of inhibitory receptors such as PD-1 and cytotoxic T lymphocyte–associated antigen 4. Although CTLs are a prominent component of the tumor microenvironment (TME), they are rendered largely ineffective in tumor clearance in glioma.

Prior work has shown that suboptimal antigen presentation and poor priming of CTLs by tumor cells are causally linked to anergic and exhausted T-cell phenotypes (20, 21). A low mutational burden in glioma significantly restricts the expression of cancer-specific neoantigens as compared with other cancers (22, 23). Accordingly, vaccine-based approaches against glioma antigens including targeted approaches toward tumor-specific antigens like EGFRvIII or personalized neoantigen approaches have not yielded successful clinical improvement (24, 25). The role of antigen presentation in glioma-associated immunosuppression and tumor progression remains poorly defined.

To examine the role of antigen presentation by glioma we queried our human glioma single-cell RNA sequencing (scRNA-seq) dataset and identified a rare population of tumor cells with elevated expression of CD83, a marker of mature antigen-presenting cells (APC) that normally acts to prolong CD8^+^ T-cell expansion and promotes CTL-mediated antitumor immunity (26–28). Here, we show that overexpression of CD83 in an immunocompetent glioma model extends survival and is associated with activation and expansion of CD8^+^ T cells while enhancing activating T-cell cytokine production. Our results show that CD83 expression in glioma leads to expression profiles related to CTL-mediated antitumor responses and highlight novel mechanisms by which tumor cells could partake in priming of T cell–mediated immunity in glioma.

## Materials and Methods

### Ethics statement

The experiments conducted were approved by the institutional review board at Baylor College of Medicine. Approval for analysis of human tissue was granted by protocol H35355. Approval for mouse experiments was granted by Institutional Animal Care and Use Committee (IACUC) AN6100.

### Sex as a biological variable

All experimental animals were treated in compliance with the US Department of Health and Human Services and Baylor College of Medicine IACUC guidelines. All mice were housed with food and water available *ad libitum* in a 12-hour light/dark environment. Both male and female mice were randomly allocated to experimental groups. All scRNA-seq studies were performed on mice of the same sex. Adult mice older than 3 months were used for *ex vivo* and *in vivo* experiments unless otherwise stated. Adult patients at St. Luke’s Medical Center and Ben Taub General Hospital provided preoperative informed consent to participate in the study and gave consent under Institutional Review Board Protocol H35355. Patients included males and females. Clinical characteristics were maintained in a deidentified patient database and are summarized in Supplementary Table S1.

### Human data

Tumor samples were collected during surgery and immediately placed on ice. Tissue was divided for use in subsequent transcriptomic, histopathologic, proteomic, or biochemical studies. Patient samples were collected separately for pathology and molecular subtyping. Histopathology and molecular subtyping of isocitrate dehydrogenase (*IDH*) and 1p19q deletion status were confirmed by board-certified pathologists.

### piggyBac *in utero* electroporation model

Tumor mice were generated according to previously published protocols (29). Briefly, *in utero* electroporation and single-sided intraventricular injection of *Pten*, *Nf1*, and *Trp53* CRISPR/Cas9 pX330 constructs targeting *Glast*-expressing mouse neural precursor cells via piggyBac transposase technology were performed on CD1 wild-type (WT) damns at embryonic day 16.5. The sgRNA guides used for these experiments were as follows:

Δ*Pten* sgRNA: GAGATCGTTAGCAGAAACAAAAGG
Δ*NfI* sgRNA: GCAGATGAGCCGCCACATCGAGGG
Δ*Trp53* sgRNA: CCTCGAGCTCCCTCTGAGCCAGG
Δ*Cd83*–*1* sgRNA: CTGCAGCCTGGCACCCGCGA
Δ*Cd83–2* sgRNA: CTTGGCCCAGGACACTGCAT

CD83^OE^ tumor mice were generated using piggyBac constructs driving overexpression of mouse *Cd83*. All mice underwent coelectroporation of piggyBac-GFP constructs. Tumor brains were collected from mice either at matched timepoints or end-stage disease. Mice were monitored for symptoms indicative of tumor burden, including lethargy, hunched posture, decreased appetite, poor grooming maintenance, squinting of the eyes, partial limb paralysis, or ataxia, denoting the IACUC-permitted endpoint.

### Single-cell processing

We ran samples on the 10X Chromium platform to produce next-generation sequencing libraries. We followed standard procedures, using the Seurat pipeline, for filtering, removing mitochondrial genes, and selecting variable genes. Our criteria for including cells and genes were as follows: genes present in more than three cells, cells expressing more than 300 genes, cells with a gene count ranging from 200 to 5,000, and a mitochondria ratio of 10 (or less than 20 in mice). To integrate cells from different patients or mice experiments, we used the Harmony algorithm (30). Next, we visualized clusters using a uniform manifold approximation and projection constructed from the principal component analysis (PCA) corrected by Harmony. This visualization was created using the *runUMAP*, *FindNeighbors*, and *FindClusters* functions of the Seurat package. We extracted differentially expressed genes (DEG) among clusters using *FindAllMarkers* function of Seurat package. Seurat performs differential expression testing based on the nonparametric Wilcoxon rank-sum test and applies Bonferroni correction to adjust for multiple testing.

### scRNA-seq

Human and GFP^+^ mouse tumors were prepared as single-cell suspensions. Briefly, samples were coarsely chopped with surgical scissors and enzymatically digested with papain supplemented with DNase I (Worthington Biochemical Corporation, LK003150). Samples were incubated for 15 minutes at 37°C on a thermocycler kept at 1,400 × *g* and briefly pipetted every 5 minutes. Cells were pelleted at 13,000 × *g* for 10 seconds and resuspended in PBS before processing for debris and dead cell removal. Cell suspensions were processed using the MACS Debris Removal Kit (Miltenyi, 130-109-398) and MACS Dead Cell Removal Kit (Miltenyi, 130-090-101), according to the manufacturer’s instructions. Live cells were collected through negative selection using an MS Column in the magnetic field of a MiniMACS Separator (Miltenyi, 130-042-102). Eluted cells were spun at 300 × *g* for 5 minutes and resuspended in Gibco DMEM with GlutaMAX (Thermo Fisher Scientific, 10566016) supplemented with 10% FBS (Thermo Fisher Scientific, 16000044). Single cells were processed with the 10X Chromium 3′ Single-Cell Platform using the Chromium Single-Cell 3′ Library, Gel Bead, and Chip Kits (10X Genomics) following the manufacturer’s protocol. Briefly, approximately 5,000 to 15,000 cells were added to each channel of a chip to be partitioned into Gel Beads in Emulsion in the Chromium instrument, followed by cell lysis and barcoded reverse transcription of RNA in droplets. Gel Beads in Emulsion were broken, and cDNA from each single cell was pooled. Clean-up was performed using Dynabeads MyOne Silane Beads (Thermo Fisher Scientific, 37002D). Subsequently, the cDNA was amplified and fragmented to optimal size before end repair, A-tailing, and adapter ligation. Libraries were run individually using a NextSeq 500/550 High Output Kit v2.5 (75 Cycles; Illumina, 20024907) and sequenced on an Illumina NextSeq550 instrument.

### Histology

Mice were humanely euthanized, and brain tissue was harvested for subsequent processing. Mouse brain samples were fixed through intracardial perfusion of 4% paraformaldehyde in PBS and kept in solution for 12 hours at 4°C before being transferred to 70% EtOH. Human samples were drop-fixed in 4% paraformaldehyde in PBS for 12 hours at 4°C before being transferred to 70% EtOH. Paraffin embedding was performed by the Breast Cancer Pathology Core at Baylor College of Medicine.

Hematoxylin and eosin staining was performed on 10-μm paraffin-embedded sections cut on a microtome. Slides were deparaffinized and processed using Harris hematoxylin (Poly Scientific R&D, S212A) and eosin (Poly Scientific R&D, S176) for 1 minute and 30 seconds, respectively. Slides were mounted with Permount mounting medium (Electron Microscopy Sciences, 17986-01) before coverslips were placed. Histologic diagnoses of mouse tumors were validated in at least three tumors per experimental group by a board-certified neuropathologist.

For immunohistology, 10-μm paraffin-embedded sections were cut, deparaffinized, and subjected to heat-induced epitope retrieval using antigen retrieval buffer (10 mmol/L sodium citrate, 0.05% Tween 20, pH 6.0). Sections were blocked for 1 hour at room temperature and incubated in primary antibodies (goat anti-CD83; 1:200; Novus Biologicals, AF1437) overnight at 4°C. Species-specific secondary antibodies tagged with Alexa Fluor corresponding to 568-nm emission spectra (1:1,000, Thermo Fisher Scientific) were used for immunofluorescence. Hoechst nuclear counterstaining (1:50,000; Thermo Fisher Scientific, H3570) was performed before coverslips were mounted using Vectashield antifade mounting medium (Vector Laboratories, H-1000).

### Annotation of tumor cells in human scRNA-seq data

We used multiple orthogonal tumor features to identify individual tumor cells in three modular components: (i) marker expression modeling, (ii) genotyping of copy number variations (CNV) of all cells, and (iii) RNA-inferred mutational profiling of known glioma mutations (i.e., *IDH* and *EGFR*). These modules are described below in more detail.

a. *Marker expression–based tumor classification model*

We use finite Gaussian mixture modeling to accurately depict the expression of three key tumor marker genes: *SOX2*, *EGFR*, and *PDGFRA*. This modeling approach enables the discrimination of tumor cells based on their distinct marker gene expression profiles.

We used previously published datasets of both tumor and nontumor cells to establish a model for classifying tumor cells based on marker expression (i.e., determining “high expression” thresholds) for the tumor markers *PDGFRA*, *EGFR*, and *SOX2* (31, 32). For each tumor marker, we constructed an independent classifier model using (i) the Allen Brain Atlas (31) human scRNA-seq data as a training set for normal cells, representing a comprehensive collection of healthy brain data, and (ii) a compendium of publicly available brain tumor scRNA-seq datasets as a training set for tumor cells (32). We utilized statistical models, specifically a mixture of Gaussian distributions, to infer the class (normal vs. tumor) of our in-house tumor scRNA-seq data based on marker expression.

To identify and classify normal and tumor cells based on expression, we employed a mixture of Gaussian distributions. We utilized a finite mixture model with *G* = 2 classes (normal vs. tumor) to define the probability density function for each expression value. We applied this model to predict the “normal” vs. “tumor” class in our in-house glioma cells. For each gene, the posterior probabilities were estimated by maximizing the log-likelihood function through the expectation–maximization algorithm. For each tumor marker, we generated a matrix with genes indicated by rows and cells indicated by columns. The cell value index was set to 1 if the cell had a high “tumor class” probability for the corresponding gene. We implemented the Gaussian mixture model using the mclust R package (33).

b. *RNA-inferred genotyping of chromosome alterations*

We incorporated our CNV-calling algorithm, CaSpER (34), as well as the state-of-the-art CNV-calling method numbat (35).

c. *RNA-inferred mutational profiling*

For deducing rare deleterious single-nucleotide variants (SNV) present in tumor cells, we used our recently developed tool called XCVATR (36). This analysis involves considering SNVs that are documented in the COSMIC (37) database and have a frequency of less than 0.1% in the dbSNP (38) database.

These distinct tumor features described above are called independently within our analysis. By integrating these different approaches, we aim to accurately identify tumor cells at a single-cell resolution.

To classify a single cell as a “tumor” within our framework, it must meet one or more of the following criteria:

1. Marker expression modeling: The expression levels of at least one tumor cell marker (*SOX2*, *EGFR*, or *PDGFRA*) must exceed a predetermined threshold. This threshold is determined using finite Gaussian mixture modeling, as explained in the above section “Marker Expression–based tumor classification model.”
2. RNA-inferred genotyping of chromosome alterations: The presence of large-scale CNV is indicative of a tumor cell.
3. RNA-inferred mutational profiling: Tumor cells that possess SNVs in the genes *IDH* (R132H/R132C) or *EGFR* are considered as tumor cells.

### Annotation of tumor cells in mice scRNA-seq data

We used the expression levels of at least one tumor cell marker (*Sox2*, *Egfr*, or *Pdgfra*) to annotate tumor cells in mouse data. To be classified as a tumor, gene expression must exceed a predetermined threshold determined by the %99 quantile of gene expression values in the Allen Brain Mouse Atlas (*Pdgfra* > 2.64, *Egfr* > 2.04, *Sox2* > 1.95) or expresses *GFP* > 0.

### Generating CD83 gene set

To generate Cd83^+^ tumor signature, we performed a comparison between two groups of tumor cells. The first group consisted of tumor cells that are Cd83^+^ (expressing Cd83) and Cd45^−^ (lacking Cd45 expression) in Cd83 gain-of-function (GOF) experiment mice. The second group consisted of tumor cells that are Cd83^−^ and Cd45^−^ originated from WT mice. We used the logFC > 1 and adjusted *P* value < 0.05 threshold in generating the DEGs. We next converted Cd83^+^ tumor signature mouse symbols to their human counterparts and assessed the importance of the expression of this gene set in patient survival outcomes.

### Immune gene set scoring in single-cell data

We scored individual tumor cells obtained in our human scRNA-seq dataset using our CD83 gene set and 130 previously published immune gene sets (39, 40) using the Seurat *AddModuleScore* function. To analyze the relationship between the CD83 gene set score and the scores of the 130 immune gene sets, we calculated Pearson correlation coefficients for each sample. The statistical significance of these correlations was determined using the Pearson correlation test.

### Immune gene set scoring on pseudobulked tumor single cells

We aggregated tumor cells from each single-cell sample into pseudobulk by summing gene counts across all tumor cells within each sample. Using the GSVA R package, we then performed single-sample gene set enrichment analysis (ssGSEA). Finally, we calculated the correlation between the CD83 gene set score and the 130 immune gene set scores for each pseudobulked sample. The statistical significance of these correlations was determined using the Pearson correlation test.

### Analyzing bulk expression data and survival analysis

We obtained the Cancer Genome Atlas high-grade glioma (TCGA-GBM) and TCGA-low-grade glioma (LGG) raw read counts and clinical data using the TCGAbiolinks R package (41). The TCGA-GBM, TCGA-LGG, and our bulk RNA-seq data of the IDH Mutant cohort were both normalized and applied variance stabilizing transformation using the DESeq2 package (42). To assess the gene set enrichment in human data, we performed iterative ssGSEA using the GSVA R package. In this process, genes from the CD83 gene set with expression correlations below 0.5 to the enrichment score were filtered out. We applied iterative ssGSEA because the CD83 gene set was derived from mouse single-cell data and required refinement for human data. To stratify the bulk RNA-seq data, we used the CD83 gene set scores derived from the ssGSEA analysis with the GSVA R package. The scores were computed for each sample to represent the enrichment of the CD83 gene set. The ssGSEA CD83 gene set scores were split by the upper and lower quartiles to assign samples to the high-cd83 and low-cd83 groups. This stratification allowed us to compare the impact of CD83 gene set scores on clinical outcomes. We assessed the relationship between these stratified gene set scores and overall survival using a Kaplan–Meier survival model. To evaluate survival differences between the high-cd83 and low-cd83 groups, we applied the log-rank test to calculate the *P* value. For this analysis, we utilized the survminer and survival R packages.

### CellChat cell-type interaction analysis

We applied the CellChat pipeline to infer cell–cell interactions between cell types in human and mice scRNA-seq datasets. Specifically, we compared interactions among T cells, tumor, and immune cells in mice GOF versus WT and between high CD83-scoring human samples (OG-01_Core, DA-01_Core, and OG-02_Core) and low CD83-scoring human samples (GBM-07_Core, GBM-02_Core, and GBM-01_Core). First, we identified cell type–specific communication in the mice GOF and WT groups, as well as in the high and low CD83 human sample groups, separately. We then used CellChat to compare the total number of interactions and interaction strength across the inferred cell–cell communication networks in the human and mice scRNA-seq datasets. The number of cell-type interactions was calculated using the *compareInteractions* function, and differential interactions were visualized with the *netVisual_diffInteraction* function. Finally, we used the *rankNet* function to identify and visualize conserved and context-specific signaling pathways.

### *In vivo* cell proliferation assay

Tumor proliferation was assessed through quantitative IHC using rabbit anti-KI67 (1:200; Abcam, ab16667). In total, 27 bright-field images were acquired per experimental group (*n* = 3 images per coronal section × *n* = 3 sections per animal × *n* = 3 animals per experimental group). Quantification was performed using the Analyze Particles plugin in ImageJ.

### Mouse tumor cell lines

Tumor cell lines from control, CD83^OE^, and CD83^KO^ tumors were established from P65 pB-IUE tumor mice. Mice were humanely euthanized, and the brains were dissected. GFP-guided microdissection of tumor tissue was performed under a dissection microscope. The tissue was processed into single-cell suspensions according to our scRNA-seq protocol and seeded into T75 flasks in Gibco DMEM with GlutaMAX (Thermo Fisher Scientific, 10566016) supplemented with 10% FBS (Thermo Fisher Scientific, 16000044). Cells were grown for 2 weeks (passaged at 85% confluency) before use in subsequent assays. Tumor cell lines were validated using a Surveyor Assay for the *Pten*, *Trp53*, *Nf1*, and *Cd83* insertions/deletions, according to the manufacturer’s instructions (IDT, 1075932). The following primers were used for PCR amplification of insertions/deletions–containing loci:

*Trp53* forward GCT​TTC​CCA​CCC​TCG​CAT​AA
*Trp53* reverse TCA​CAC​GAA​AGA​CAA​CTC​CCC
*Nf1* forward TCT​GTA​CCT​CTT​GGA​CTA​TGC​C
*Nf1* reverse TGA​GCC​TCA​AAA​CTT​GCT​TGG
*Pten* forward AGG​ATT​ATC​CGT​CTT​CTC​CCC​A
*Pten* reverse ACC​CTC​AAA​TGT​GCA​CCG​TC
*Cd83* forward CCAAGCGCGGGTACAAGA
*Cd83* reverse CTC​TCT​CAG​AAC​CTC​GCT​GA

### *In vitro* cell proliferation assay

*In vitro* cell proliferation was assessed in mouse tumor cell lines using the Click-iT EdU Assay (Thermo Fisher Scientific, C10340), according to the manufacturer’s guidelines. Briefly, 6 × 10^4^ cells were seeded onto 12-mm poly-D-lysine–coated coverslips in a 12-well culture dish. After 48 hours, cells were pulsed with EdU for 2 hours before being fixed with 4% paraformaldehyde and processed for immunostaining. Quantification was performed using 27 images per experimental group (*n* = 3 images per coverslip × *n* = 9 coverslips per experimental group). EdU positivity was assessed as a fraction of the total cells using Hoechst counterstaining. Images were processed using the Analyze Particles plugin in ImageJ.

### Coculture experiment

Mouse tumor cell lines were seeded into T25 flasks and grown to 50% confluency. Naïve CD8^+^ T cells were harvested from CD1 WT P25 mice and processed using the EasySep Mouse Naïve CD8^+^ T Cell Isolation Kit (STEMCELL Technologies, 19858). Following isolation, 10 × 10^5^ naïve CD8^+^ T cells were seeded onto mouse tumor cell lines and maintained in RPMI 1640 media (Thermo Fisher Scientific, 11875093) supplemented with 10% FBS (Thermo Fisher Scientific, 16000044) and 1% penicillin–streptomycin solution. Cells were left in coculture for 1 week before the T cell–containing medium was harvested for use in imaging flow cytometry experiments.

### Multispectral imaging flow cytometry

For CD83 experiments, GFP^+^ tumor tissue from control pB-IUE tumor mice at postnatal day 65 was processed into a single-cell suspension. Briefly, cells were centrifuged at 400 × *g* for 3 minutes at 4°C and washed three times in MACS Buffer (Miltenyi Biotec, 130-091-222) supplemented with 4% FBS (Thermo Fisher Scientific, 16000044) before being incubated with APC-conjugated rat anti-CD83 antibody (1.25 μL/10 × 10^6^ cells; BioLegend, 121510) for 30 minutes on ice. Cells were centrifuged at 400 × *g* for 3 minutes at 4°C and washed three times in MACS Buffer before being resuspended in 50 μL MACS Buffer for imaging experiments. High expression of endogenous GFP was used to gate tumor cells.

Image cytometry assays were acquired on an ImageStream X MKII (Luminex) equipped with a 405-, 488-, 561-, 633-, and 785-nm scatter laser. Collection was performed on as many objects as feasible in 2 hours. The number of objects collected ranged from 100,000 to 300,000 to allow for the analysis of rare CD83^+^ events. Objects were analyzed and gated using IDeaS software 6.3.23.0. Cells were gated using the Aspect Ratio and Area parameters for the bright-field channel. Focused cells were gated using the Gradient_RMS parameter for channel 1 (Brightfield). CD83^+^GFP^+^ cells were gated by signal intensities for channel 2 (GFP) and channel 11 (CD83 APC).

For T-cell experiments, T cell–containing medium was harvested from cocultures, centrifuged at 700 × *g* for 3 minutes at 4°C, and washed three times in MACS Buffer before incubation with the following antibodies: APC-conjugated rat anti-CD3 (5 μL/10 × 10^6^ cells; BioLegend, 100408), PE-conjugated rat anti-CD8b (2.5 μL/10 × 10^6^ cells; BioLegend, 126608), PE/Cyanine7-conjugated rat anti-CD25 (2.5 μL/10 × 10^6^ cells; BioLegend, 101916), and Brilliant Violet 421-conjugated rat anti-CCR7 (5 μL/10 × 10^6^ cells; BioLegend, 120120). The cells were incubated for 30 minutes on ice, centrifuged at 700 × *g* for 3 minutes at 4°C, and washed three times in MACS Buffer. The cells were resuspended in Cytofix/Cytoperm Solution (Thermo Fisher Scientific, 00-5523-00), incubated for 30 minutes at 4°C in the dark, and washed three times before being resuspended in 50 μL MACS Buffer for imaging experiments. Twenty-thousand cells were acquired based on the Aspect Ratio versus Area of Brightfield. Data were analyzed by gating on focused, single cells, followed by channel 2 (CD8 PE) and channel 11 (CD3 APC) to determine CD3^+/−^CD8^+/−^ cells. These were then analyzed using the channel 7 (CCR7 BV421) and channel 6 (CD25 PE-Cy7) intensities.

Representative images used for figures were exported from the IDeaS image gallery as .tif files and inserted into the article. Raw data files are available upon request.

### ELISA

Medium was collected from mouse tumor cells cocultured with naïve CD8^+^ T cells for 1 week or without T cells. Cells were maintained in T25 tissue culture flasks with RPMI 1640 medium (Thermo Fisher Scientific, 11875093) supplemented with 10% FBS (Thermo Fisher Scientific, 16000044) and 1% penicillin–streptomycin solution. Harvested medium was centrifuged at 3,000 × *g* for 15 minutes to remove debris and cells. The supernatant was collected and used for subsequent ELISA experiments. The following ELISA kits were used according to the manufacturer’s instructions: mouse IFNγ Quantikine ELISA kit (R&D Systems, MIF00), mouse TNFα Quantikine ELISA kit (R&D Systems, M2000), and mouse IL-2 Quantikine ELISA kit (R&D Systems, M2000). For statistical analyses, technical replicates (*n* ≥ 14) were taken per experimental condition.

### Statistical analysis

The following tests were used for statistical analysis unless otherwise noted. For Kaplan–Meier survival analysis, the log-rank test was used to compare survival differences among groups. For quantified results, ANOVA was used, followed by the Welch *t* test to compare individual means. For RT-qPCR, a two-tailed Student *t* test was used. Significant differences are denoted by asterisks in associated graphs. For the Cd83 immune gene set scoring expression correlation analyses, the Pearson correlation test was used. Data are presented as the mean ± SEM. Levels of statistical significance are indicated as follows: ns, not significant; *, *P* < 0.05; **, *P* < 0.01; ***, *P* < 0.001, and ****, *P* < 0.0001.

### Data availability

The data generated in this study are available upon request from the corresponding author.

All other study data are included in the article and/or supporting information.

The source code is available at https://github.com/akdess/cd83.

## Results

### Antigen presenting–like tumor cells are found in glioma

**Figure 1:**
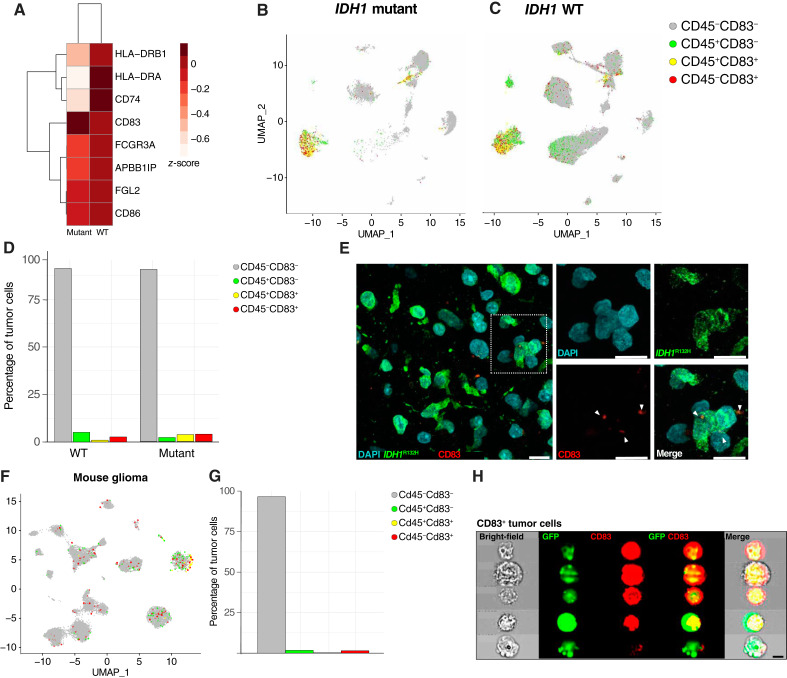
Antigen presenting–like tumor cells express Cd83 in human and mouse glioma. Expression of GO:0002291: T-cell activation via T-cell receptor contact with antigen bound to MHC molecule on APC and GO:0002468: dendritic cell antigen processing and presentation in human IDH^WT^ and IDH^mut^ glioma tumor cells that were differentially expressed (**A**). scRNA-seq from five IDH^mut^ and seven IDH^WT^ gliomas demonstrate a rare population of CD83^+^ tumor cells (**B** and **C**). Percentage of IDH^mut^ and IDH^WT^ tumor cells from scRNA-seq that expressed different combinations of CD45 and CD83 (**D**). Immunofluorescence imaging for *IDH1*^R132H^ tumor cells that express CD83 (**E**). scRNA-seq from pB-IUE glioma isolates similar murine tumor cells (**F**). Percentage of murine tumor cells from scRNA-seq that expressed different combinations of CD45 and CD83 (**G**). Multispectral flow cytometry isolates a distinct population of GFP^+^CD83^+^ tumor cells (**H**). (Created with Biorender.com.)

Leveraging our scRNA-seq dataset of 234,880 cells collected from patients with IDH WT (IDH^WT^; *n* = 7, grade IV) and mutant (IDH^mut^; *n* = 5, grades II–IV) glioma (29), we annotated each cell as tumor or nontumor based on the presence of CNVs and/or SNVs in tumor suppressor genes (see “Materials and Methods”; Supplementary Fig S1A–S1K). Our IDH^mut^ samples contain a mixture of histopathologic diagnoses and grades that were analyzed in parallel to the IDH^WT^ GBM samples. To determine whether tumor cells express antigen-presenting and/or T cell–activating genes, we examined the expression of 13 genes from two gene ontologies (GO:0002291 and GO:0002468) related to T-cell antigen presentation in our human glioma scRNA-seq dataset. Of those 13 genes, eight were differentially expressed between IDH^WT^ and IDH^mut^ tumor cells (Fig. 1A). Of the 13 genes queried, we found that *CD83* was the only gene for which expression was high in both IDH^mut^ and IDH^WT^ tumor cells, so we focused our studies on CD83 expression in glioma cells. Despite the varied outcomes and grades of our tumor samples, our analysis of CD83^+^ tumor cells revealed that a rare cell population is present in both IDH^mut^ (7.7%) and IDH^WT^ (3.78%) tumors (Fig. 1B–D). Many phagocytosing immune cells actively surveil the TME (43), therefore to ensure CD83^+^ tumor cells were not an artifact of this process, we assessed CD83^+^ tumor cells that did not express the immune lineage marker CD45. Immunostaining for *IDH1*^R132H^ and CD83 in an IDH^mut^ patient sample confirmed the existence of these CD83^+^CD45^–^ tumor cells, which we termed antigen presenting–like tumor cells (ALT; Fig. 1E).

Next, we extended these observations to our *in utero* electroporation (IUE) mouse model of high-grade glioma, identifying an analogous population of ALTs using our existing scRNA-seq dataset (1.7% of GFP^+^ tumor cells; Fig. 1F and G; Supplementary Fig. S2A and S2B). Exploiting the GFP-labeling of IUE-based glioma cells, we employed multispectral imaging flow cytometry and identified a population of CD83^+^GFP^+^ ALTs that were characterized by high, diffuse intracellular GFP expression, a hallmark of targeted cells in the pB-IUE model (Fig. 1H; Supplementary Fig. S3A–S3C; ref. 24). Collectively, these analyses identified ALTs as a rare population of tumor cells in both human and mouse glioma that possess high expression of CD83, a key mediator of the antigen presentation process.

### CD83 suppresses tumor progression

**Figure 2:**
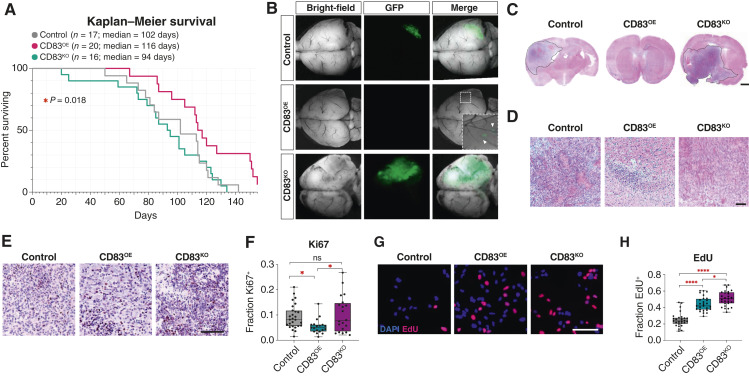
CD83 influences tumor growth in the pB-IUE system. Kaplan–Meier survival curve of pB-IUE tumors control (*n* = 17, Med OS = 102 days), CD83^KO^ (*n* = 20, Med OS = 94 days), and CD83^OE^ (*n* = 16, Med OS = 116 days; log-rank, all three curves, *P* value = 0.018; **A**). Age-matched P60 brains demonstrating decreased growth of CD83^OE^ tumors compared with control and CD83^KO^ tumors (**B**). Hematoxylin and eosin stain of pB-IUE control, CD83^OE^, and CD83^KO^ tumors (**C** and **D**). IHC for Ki67 of pB-IUE control, CD83^OE^, and CD83^KO^ tumors (one-way ANOVA *P* value = 0.0133. Tukey MCT: CD83^KO^ vs. CD83^OE^*P* value = 0.0158, CD83^KO^ vs. control *P* value = 0.8075, and CD83^OE^ vs. control *P* value = 0.0454; **E** and **F**). EdU staining of *in vitro* cultured control, CD83^OE^, and CD83^KO^ tumors (one-way ANOVA *P* value < 0.0001. Tukey MCT: CD83^KO^ vs. CD83^OE^*P* value = 0.0193, CD83^KO^ vs. control *P* value < 0.0001, and CD83^OE^ vs. control *P* value < 0.0001; **G** and **H**). *, *P* value < 0.05; ****, *P* value < 0.0001. (Created with Biorender.com.)

Having identified ALTs in human and mouse glioma, we sought to define how CD83 expression affects *in vivo* tumor progression. We used CRISPR/Cas9 technology to generate CD83 loss-of-function (CD83^KO^) IUE-based mouse tumors and separately used murine *Cd83* overexpression to generate CD83 GOF (CD83^OE^) tumors (Supplementary Fig. S3D). CD83^KO^ tumors were associated with a modest reduction in survival times (Med OS = 94 days vs. 102 days). Alternatively, CD83^OE^ tumors were associated with an increase in survival times compared with controls (Med OS 116 days vs. 102 days; Fig. 2A).

To determine how CD83 expression influences tumor features, we assessed age-matched P60 CD83^OE^, CD83^KO^, and control tumors. Gross examination of tumors using GFP^+^ expression revealed that CD83^OE^ tumors were smaller and that CD83^KO^ tumors were larger than control tumors (Fig. 2B). Histopathologic analysis revealed features consistent with high-grade gliomas in both control and CD83^KO^ tumors, whereas CD83^OE^ tumors showed smaller regions of hyperproliferating cells without necrosis and microvascular proliferation (Fig. 2C and D). Analysis of tumor proliferation via Ki67 revealed no difference in proliferation rates between CD83^KO^ and control tumors, whereas CD83^OE^ tumors demonstrated significantly reduced proliferative capacities (5.5% Ki67 CD83^OE^, 10.27% Ki67 CD83^KO^, 9.3% Ki67 control, one-way ANOVA *P* value = 0.0133. Tukey MCT: CD83^KO^ vs. CD83^OE^*P* value = 0.0158, CD83^KO^ control *P* value = 0.8075, and CD83^OE^ vs. control *P* value = 0.0454; Fig. 2E and F). To determine if the effects of CD83 expression on tumor growth are reliant upon extrinsic cues, we derived cell lines from tumor-bearing mice and assessed proliferation *in vitro* with an EdU assay. In contrast to *in vivo* experiments, both CD83^OE^ and CD83^KO^ tumor cells showed increased proliferative capacities *in vitro* (44.2% EdU^+^ CD83^OE^, 51% EdU^+^ CD83^KO^, 25.1% EdU^+^ control, one-way ANOVA *P* value < 0.0001. Tukey MCT CD83^KO^ vs. CD83^OE^*P* value = 0.0193, CD83^KO^ vs. control *P* value < 0.0001, and CD83^OE^ vs. control *P* value < 0.0001; Fig. 2G and H). These results suggest that modulation of CD83 *in vitro* in tumor cells could have cell-intrinsic effects leading to increased ALT proliferation. However, in the CD83^OE^ tumors, these effects seem to be mitigated by cell-extrinsic mechanisms that suppress CD83-induced proliferation to slow tumor progression *in vivo*.

### ALTs are associated with antitumor CD8^+^ T-cell profiles in glioma

**Figure 3:**
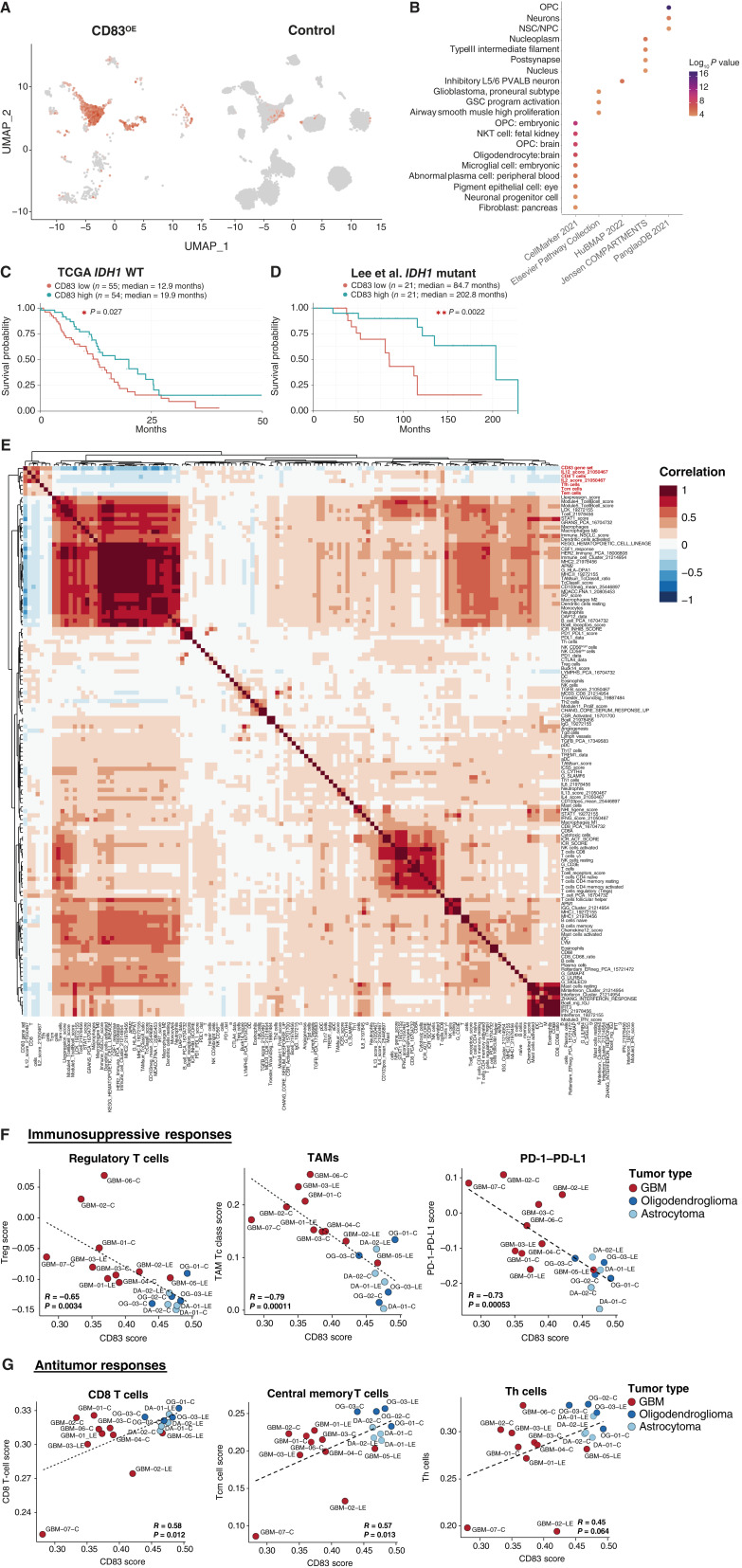
CD83 gene signature related to tumor survival, reduced immunosuppressive response signatures, and enhanced antitumor response gene signatures. Expression of CD83 in scRNA-seq from pB-IUE CD83OE compared with control pB-IUE tumors (**A**). EnrichR analysis of the identified DEGs enriched in CD83^+^ tumor cells (**B**). Kaplan–Meier survival of TCGA IDH^WT^ GBM of tumors with high CD83 gene scoring compared with low CD83 gene scoring (median = 19.9 months vs. 12.9 months, *P* value = 0.027; **C**). Kaplan–Meier survival of IDH^mut^ low-grade gliomas with high CD83 gene scoring compared with low CD83 gene scoring (median OS = 202.8 months vs. 84.7 months, *P* value = 0.0022; **D**). Heatmap of CD83 gene scoring and immune expression gene profiles (**E**). Immunosuppressive gene sets vs. CD83 gene score showing an inverse relationship between CD83 signature and regulatory T cell (*R* = −0.65; *P* = 0.0034), TAM (*R* = −0.79; *P* = 0.00011), and PD-1–PD-L1 (*R* = −0.73; *P* = 0.00053) gene signatures in our 12 human glioma single-cell samples (**F**). Antitumor response gene sets vs. CD83 gene score showing a direct relationship between CD83 signature and CD8 T cell (*R* = 0.58; *P* = 0.012), central memory T cell (*R* = 0.57; *P* = 0.013), and Th cell (*R* = 0.45; *P* = 0.064) gene signatures in our 12 human glioma single-cell samples (**G**). *, *P* value < 0.05; **, *P* value < 0.01. (Created with Biorender.com.)

To dissect how CD83 affects tumor growth and the associated immune microenvironment, we performed scRNA-seq on CD83^OE^ and CD83^KO^ tumors, confirming a significant 2.98 log_2_ fold change increase (adjusted *P* value < 2.2e−16) in CD83 expression within CD83^OE^ tumor cells when compared with control tumor cells (Fig. 3A; Supplementary Fig. S3E). To identify the transcriptional features imparted onto tumor cells by the expression of CD83, we compared CD83^+^CD45^–^ tumor cells from CD83^OE^ tumor mice to CD83^–^CD45^–^ tumor cells from control tumor mice using DEG analysis (Supplementary Table S2). EnrichR analysis of the identified DEGs (log_2_ fold change > 1; adjusted *P* value < 0.05) showed that most genes enriched in CD83^+^ tumor cells relate to neural and glioma cell pathways (Fig. 3B), indicating that expression of CD83 in tumor cells imparts biologically relevant changes to glioma cell transcriptomes. These transcriptomes captured the uniqueness of CD83 tumor cells showing a combination of primitive glioma-like cell subtypes such as oligodendrocyte progenitor cell and neural progenitor cell as well as antigen-presenting cells like embryonic microglia. We used this DEG set to stratify bulk RNA-seq samples from TCGA and an internal cohort into high and low expressors of the CD83 gene set, finding that overall survival was significantly extended in both IDH^WT^ [high CD83: 19.9 months (median) vs. low CD83: 12.9 months (median), *P* value = 0.027, log-rank] and IDH^mut^ patients [high CD83: 202.8 months (median) vs. low CD83: 84.7 months (median), *P* value = 0.0022, log-rank; Fig. 3C and D]. These collective studies suggest that the expression of CD83 by tumor cells confers a unique transcriptional signature, the effects of which are correlated with better survival outcomes in both human patients and mouse models of the disease.

Given the key role of CD83 in modulating tumor–immune interactions, we sought to define which immune populations are impacted by CD83 expression in tumor cells. Analysis of our scRNA-seq dataset using 130 previously published immune gene sets revealed that the CD83 tumor expression signature demonstrated a robust, positive correlation with CD8^+^ T cell– and T cell–activating cytokine gene sets (Fig. 3E; refs. 39, 40). Samples exhibiting low CD83 expression signatures exhibited a positive correlation with immunosuppressive gene sets pertaining to regulatory T cells, TAMs, and PD-1–PD-L1 interactions (Fig. 3F), suggesting that low levels of CD83 expression by tumor cells promote immunosuppressive phenotypes. Consistent with prior reports documenting an immunosuppressive microenvironment in IDH^WT^ tumors, we found that these samples exhibited lower CD83 expression signatures, coupled with enhanced immunosuppressive gene set signatures when compared with their IDH^mut^ counterparts (Fig. 3F). Conversely, across both IDH^mut^ and IDH^WT^ tumors, expression of the CD83 gene set positively correlated with antitumor gene sets including CD8^+^ T-cell, central memory CD8^+^ T-cell, and Th cell gene sets (Fig. 3G), which was primarily enhanced in IDH^mut^ tumor samples compared with IDH^WT^ tumors. These observations suggest that CD83^+^ ALTs could influence an antitumor T-cell transcriptional response and further implicate reduced CD83 expression in tumor cells as a potential correlate of immunosuppressive phenotypes in glioma.

### ALTs activate CD8^+^ T cells

**Figure 4:**
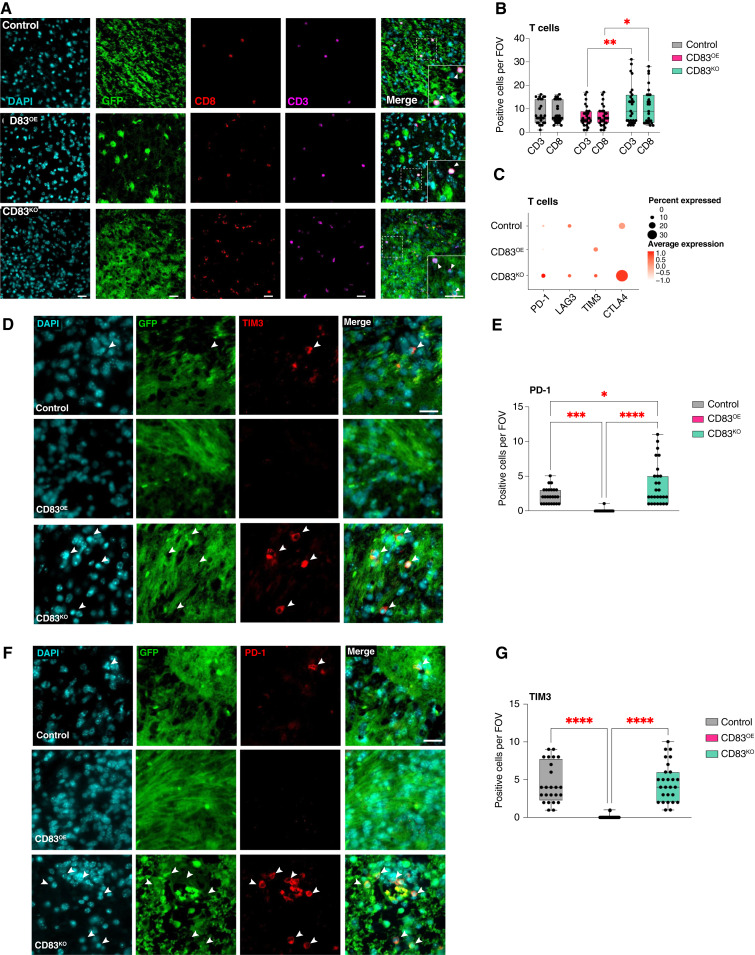
Tumoral CD83 influences expression of T-cell exhaustion markers. Immunofluorescence for CD8 and CD3 in pB-IUE control, CD83^OE^, and Cd83^KO^ tumors (**A**). Increased number of CD3 and Cd8 T cells in CD83^KO^ compared with CD83^OE^ (two-way RM ANOVA *P* value < 0.0001, tumor type *P* value = 0.0183. Tukey MCT CD3: CD83^KO^ vs. CD83^OE^*P* value = 0.0094, CD83^KO^ vs. control *P* value = 0.0897, and OE CD83^OE^ vs. control *P* value = 0.6658. Tukey MCT CD8: CD83^KO^ vs. CD83^OE^*P* value = 0.0270, CD83^KO^ vs. control *P* value = 0.1466, and CD83^OE^ vs. control *P* value = 0.7514; **B**). Expression of T-cell exhaustion markers in subset T-cells from scRNA-seq in pB-IUE IUE control, CD83^OE^, and CD83^KO^ tumors (**C**). Immunofluorescence for PD-1 and TIM3 in pB-IUE control, CD83^OE^, and CD83^KO^ tumors (**D** and **F**). Increased number of PD-1–positive cells in CD83^KO^ compared with control and CD83^OE^ tumors and in control compared with CD83^OE^ OE (one-way ANOVA *P* value < 0.0001. Tukey MCT: CD83^KO^ vs. CD83^OE^*P* value < 0.0001, CD83^KO^ vs. control *P* value = 0.0120, and CD83^OE^ vs. control *P* value = 0.0003; **E**). Increased number of TIM3-positive cells in CD83^KO^ compared with control and control compared with CD83^OE^ tumors and in control compared with CD83^OE^ (one-way ANOVA *P* value < 0.0001. Tukey MCT: CD83^KO^ vs. CD83^OE^*P* value < 0.0001, CD83^KO^ vs. control *P* value = 0.9737, and CD83^OE^ vs. control *P* value < 0.0001; **G**). *, *P* value < 0.05; **, *P* value < 0.01; ***, *P* value < 0.001; ****, *P* value < 0.0001. CTLA4, cytotoxic T lymphocyte–associated antigen 4. (Created with Biorender.com.)

To discern if modulation of CD83 expression in ALTs alters T-cell phenotypes in glioma, we examined CD8^+^ T-cell phenotypes in our pB-IUE mice. Across all tumor groups, nearly all CD3^+^ T cells identified were CD8^+^ (Fig. 4A and B). Intriguingly, the total number of tumor-infiltrating CD8^+^ T cells per field of view (FOV) was marginally increased in CD83^KO^ tumors as compared with CD83^OE^ (11.07 cells/FOV CD83^KO^ vs. 6.93 cells/FOV CD83^OE,^ Tukey MCT CD8: CD83^KO^ vs. CD83^OE^*P* value = 0.0270); however, examination of the transcriptional profiles of these cells using our scRNA-seq dataset revealed that T cells from CD83^KO^ tumors were likely nonfunctional, having the highest expression of exhausted T-cell markers (Fig. 4C). To validate the increased transcription of exhausted T-cell markers, we examined expression of PD-1 and TIM3 in our pB-IUE mice (Fig. 4D). We found increased number of PD-1–positive cells in the CD83^KO^ compared with both control and CD83^OE^ tumors (3.78 cells/FOV CD83^KO^ vs. 0.037 cells/FOV CD83^OE^ Tukey MCT *P* value < 0.0001, and 3.78 cells/FOV CD83^KO^ vs. 2.208 cells/FOV Control Tukey MCT: *P* value = 0.0120; Fig. 4E). In addition to PD-1, there were increased levels of TIM3 in both control and CD83^KO^ compared with CD83^OE^ (4.63 CD83^KO^ cells/FOV vs. 0.037 cells/FOV CD83^OE^, Tukey MCT: *P* value < 0.0001, and 4.5 control cells/FOV vs. 0.037 cells/FOV CD83^OE^ OE vs. Tukey MCT: *P* value < 0.0001; Fig. 4F and G). These *in vivo* studies validate the CD83 expression data, both of which are inversely correlated with the expression of T-cell exhaustion markers.

**Figure 5:**
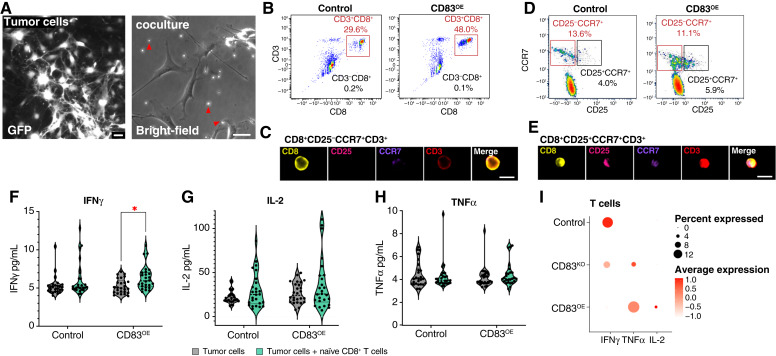
ALTs are sufficient to activate CD8^+^ T cells and act via cytokine release. *In vitro* tumor cells cocultured with isolated naïve CD8^+^ T cells (**A**). Multispectral imaging and flow cytometry for cocultured naïve CD8^+^ T cells demonstrating higher expression of mature CD3^+^CD8^+^ markers (**B–E**). ELISA for IFNγ showing increased in CD83^OE^ cocultured T cells compared with control (two-way ANOVA: tumor type *P* value = 0.7105, treatment *P* value = 0.0021, interaction *P* value = 0.6425, Šidák MCT: tumor vs. treated tumor control *P* value = 0.1160, tumor vs. treated tumor control *P* value = 0.00236; **F**). ELISA for IL-2 in control and CD83^OE^ tumors and cocultured T cells (two-way ANOVA: tumor type *P* value = 0.1314, treatment *P* value = 0.0232, interaction *P* value = 0.8087, Šidák MCT: tumor vs. treated tumor control *P* value = 0.2739, tumor vs. treated tumor control *P* value = 0.1437; **G**). ELISA for TNFα in control and CD83^OE^ tumors and cocultured T cells (two-way ANOVA: tumor type *P* value = 0.8411, treatment *P* value = 0.9121, interaction *P* value = 0.3234; **H**). Increased expression of TNFα and IL-2 by CD83^OE^ tumors compared with CD83^KO^ and control tumors in scRNA-seq from pB-IUE tumors (**I**). *, *P* value < 0.05. (Created with Biorender.com.)

Previous work has shown that coengagement of the T-cell receptor, CD28, and the ligand for CD83 supports the priming of naïve CD8^+^ T cells that retain antigen specificity and long-lasting cytotoxic function (28). To determine if ALTs can similarly engage antitumor cytotoxicity via direct priming of naïve CD8^+^ T cells, we performed coculture experiments of primary mouse glioma cell lines from control and CD83^OE^ tumors with naïve CD8^+^ T cells (Fig. 5A). Following 1 week of coculture, T cells were harvested for imaging flow cytometry, which revealed that CD83^OE^ cocultures had an increased CD3^+^CD8^+^ population (48% of live cells) as compared with controls (29.6% of live cells; Fig. 5B–E). Similar to the increased antitumor CD8 T cell response observed in the expression data, these results show that tumor cell expression of CD83 is associated with the activation of CD8^+^ T cells and might promote their expansion. These *in vitro* studies support the idea that ALT communication with CD8^+^ T cells could promote their expansion and ameliorate immunosuppressive profiles.

### ALTs support cytokine production to enhance tumor–immune communication

Because ALTs are associated with CD8^+^ T-cell expansion *in vitro*, we next examined whether activation of these cells could correlate with the acquisition of effector functions. Using ELISAs and media from our primary mouse glioma cell lines cultured alone or cocultured with naïve CD8^+^ T cells, we assessed the production of proinflammatory cytokines IL-2, IFNγ, and TNFα (44, 45). These studies revealed that CD83^OE^ cocultures demonstrated increased production of IFNγ (5.25 CD83^OE^ vs. 6.353 CD83^OE^ + T-cell, Šidák MCT: tumor vs. treated tumor control *P* value = 0.00236), with a nonsignificant increase in the production of IL-2, coupled with no changes in TNFα production (Fig. 5F–H). Analysis of T-cell profiles from CD83^OE^ tumor mice *in vivo* revealed an increase in IL-2 expression and the largest proportion of TNFα-producing cells (Fig. 5I). IFNγ production was lowest in T cells from CD83^OE^ tumor mice, suggesting that additional T-cell interactions *in vivo* likely modulate tumor-derived production of cytokines. Collectively, these data suggest that activation of CD8^+^ T cells by ALTs could induce IFNγ production *in vitro* and that T cells exposed to ALTs *in vivo* produce T-cell-activating cytokines IL-2 and TNFα.

### ALTs alter the tumor immune microenvironment

**Figure 6:**
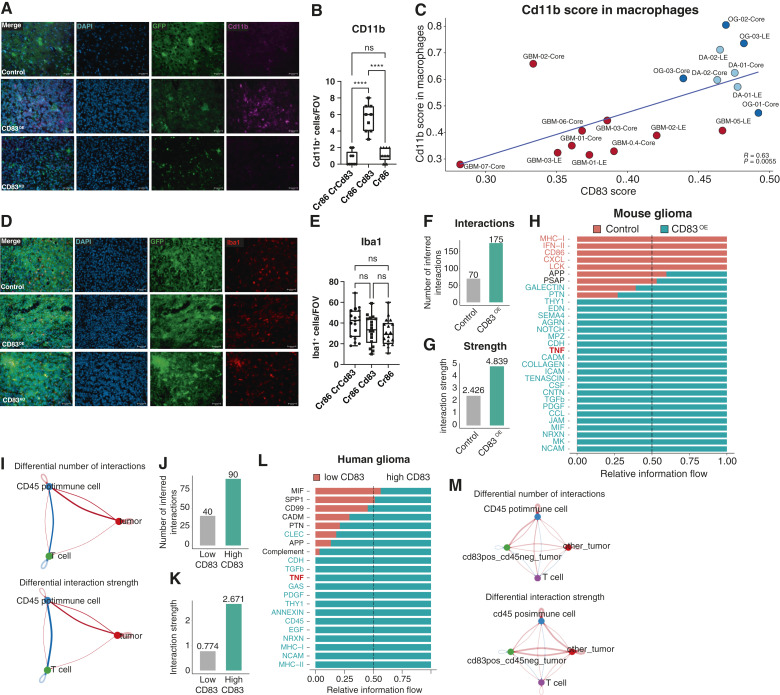
ALTs alter the myeloid compartment of the tumor immune microenvironment. Immunofluorescence for Cd11b in pB-IUE control, CD83^OE^, and Cd83^KO^ tumors (**A** and **B**; one-way ANOVA *P* value < 0.0001, Tukey MCT: Cd83^KO^ vs. CD83^OE^*P* value < 0.0001, Cd83^KO^ vs. control *P* value = 0.588, CD83^OE^ vs. control *P* value < 0.0001). Cd11b score in macrophages vs. CD83 gene scoring showing a positive relationship between CD83 signature and Cd11b score (**C**). Immunofluorescence for Iba1 in control, CD83^OE^, and Cd83^KO^ tumors (**D** and **E**; one-way ANOVA *P* value = 0.0961). CellChat comparison between CD83^OE^ and control tumor displaying number of signaling interactions (**F**), strength of the interactions (**G**), and overrepresented signaling pathways (**H**). The relative difference in number and strength of interactions within Cd45^+^ immune cells, tumor cells, and T cells between CD83^OE^ and control mouse tumor (**I**). CellChat comparison between high and low CD83 gene scoring displaying the number of signaling interactions (**J**), the strength of the interactions (**K**), and overrepresented signaling pathways (**L**). The relative difference in number and strength of interactions within Cd45^+^ immune cells, tumor cells, and T cells between high and low CD83 gene scoring human tumors (**M**). (Created with Biorender.com.)

Because ALTs were associated with increased activating cytokines that would alter other immune cells, we next sought to understand whether the tumor immune microenvironment was altered in the context of CD83^OE^. TAMs and microglia are the predominant immune constituent in gliomas (46–48). To quantify the number of monocyte cells, we stained for Cd11b in control, CD83^OE^, and CD83^KO^ pB-IUE tumors (Fig. 6A). We discovered that there were significantly more Cd11b^+^ cells in the CD83^OE^ tumors compared with control and CD83^KO^ tumors (5.56 cells/FOV CD83^OE^, 0.67 cells/FOV CD83^KO^, 1.22 cells/FOV control, 0001, Tukey MCT: Cd83^KO^ vs. CD83^OE^*P* value < 0.0001, Cd83^KO^ vs. control *P* value = 0.588, CD83^OE^ vs. control *P* value < 0.0001; Fig. 6B). Additionally, we found that Cd11b expression in macrophages significantly positively correlated with CD83 score in our human scRNA-seq (Fig. 6C). Another marker, Iba1 showed no significant differences between the populations (33.1 cells/FOV CD83^OE^, 40.4 cells/FOV CD83^KO^, 30.9 cells/FOV control, one-way ANOVA *P* value = 0.0961; Fig. 6D and E). To further dissect the signaling occurring between tumor cells, Cd45^+^ immune cells, and T cells, we utilized CellChat (49) on our mouse scRNA-seq comparing the CD83^OE^ and control tumors. We found that CD83^OE^ tumors had a greater number of interactions and a higher interaction strength, including upregulation of TNFα signaling (Fig. 6F–H). Furthermore, the interactions between both tumor cells and Cd45^+^ immune cells and tumor cells and T cells were stronger in the CD83^OE^ tumors compared with controls (Fig. 6I). We conducted a similar CellChat analysis in our human scRNA-seq and found that tumors with a high CD83 score also had increased number and strength of interactions within their cell signaling (Fig. 6J and K). We also saw increased TNFα, complement, and antigen presentation signaling occurring in high CD83-scored glioma compared with low (Fig. 6L). In tumors with high CD83, we also saw an increased number of interactions and strength of the signal between CD83 tumor cells and CD45-positive cells (Fig. 6M). Even in the CD83-negative cells tumor cells, there was increased signaling with CD45-positive immune cells and T cells in high CD83 score tumors compared with low CD83 tumors (Fig. 6M). High tumoral Cd83 expression seems to also influence the myeloid compartment of the TME.

## Discussion

Prior studies have shown that tumor cells present endogenous antigens via MHC, suggesting that MHC loss in cancer impairs naïve T-cell priming and subsequent antitumor CTL responses. Accordingly, MHC loss in glioma increases tumor invasiveness and permits immune escape, which ultimately leads to disease progression and death (50–52). Given that CD83 functions as a regulator of MHC expression and T-cell development (27, 28, 53, 54), our data introduce that it as a possible enhancer of self-antigen presentation in glioma cells, suggesting that ALTs could be promoting antitumor CTL responses by direct tumor–CTL priming of CD8^+^ T cells. Our imaging flow cytometry experiments confirmed that GFP^+^ tumor cells are actively surveilled by the surrounding immune environment, with downstream immunosuppressive responses as potential culprits for poor survival outcomes. Concordant with this hypothesis, exhausted T-cell profiles were most abundant in CD83^KO^ tumors and human patients with low expression of the CD83 gene set, which correlated with survival outcomes. Whether increased immunosuppressive transcriptional profiles of these T cells manifest in loss of cytotoxic effector functions remains unanswered and should be the focus of future inquiries.

Owing in part to the existence of a highly immunosuppressive TME, immunotherapy trials for glioma have been largely unsuccessful, showing little to no improvements in overall survival (55, 56). Additionally, standard-of-care treatments employing temozolomide, radiotherapy, and the frequent usage of dexamethasone for symptomatic relief further suppress inflammatory antitumor immunity and hinder the success of immunotherapeutic agents (57, 58). Prior studies in the field of tumor immunology have characterized numerous molecular mechanisms that underpin the immunosuppressive TME in glioma and have highlighted exhausted and anergic CTLs as contributing factors to glioma progression (59). The few immunotherapies that have shown preliminary success in eliciting systemic antitumor responses and extending survival outcomes are ultimately unable to overcome the inhibitory mechanisms employed by tumor cells (24, 60, 61). Our studies offer mechanistic insights into how tumor–CTL communication could be enhanced to potentially facilitate tumor clearance and increase survival. Our findings highlight how modulation of infrequent but potent immune constituents within the tumor could contribute to disease outcomes, demonstrating that rare tumor cell populations are not without biological consequences in human disease and should not be overlooked on the basis of scarcity.

The largest immune components in the glioma microenvironment are TAMs and microglia (46–48). We found CD83 expression affected the level of Cd11b in human and mouse cells and increased CD83 enhanced tumor–immune communications. Interestingly, one function of Cd11b (also called complement receptor 3) is as a complement receptor that mediates phagocytosis (62, 63). Agonism of Cd11b has been attempted in other cancers for antitumor efficacy and reprogramming of innate immunity (64). It is possible that the antitumor effect of CD83 tumors is mediated through complement-induced phagocytosis. We observed an increase in complement signaling in high-expressing CD83 human tumors compared with low-expressing CD83 tumors. This study suggests the possibility that tumor–T cell–myeloid interactions are enhanced by tumoral expression of CD83.

## Supplementary Material

Supplementary Table 1 Clinical characteristics

Supplementary Table 2 DEG Analysis

Supplementary Figure 1. Supplementary Figure 1

Supplementary Figure 2 Supplementary figure 2.

Supplementary Figure 3 Supplementary Figure 3.

Supplementary Data Supplemental Figure Legends
