# Supplementary figures and images for: Tumor Expression of CD83 Reduces Glioma Progression and Is Associated with Reduced Immunosuppression
Source: Cancer Res Commun. 2024 Dec 30;4(12):3209–23. doi: 10.1158/2767-9764.CRC-24-0281 (PMC11683667; doi:10.1158/2767-9764.CRC-24-0281)

# Supp. Figure 1

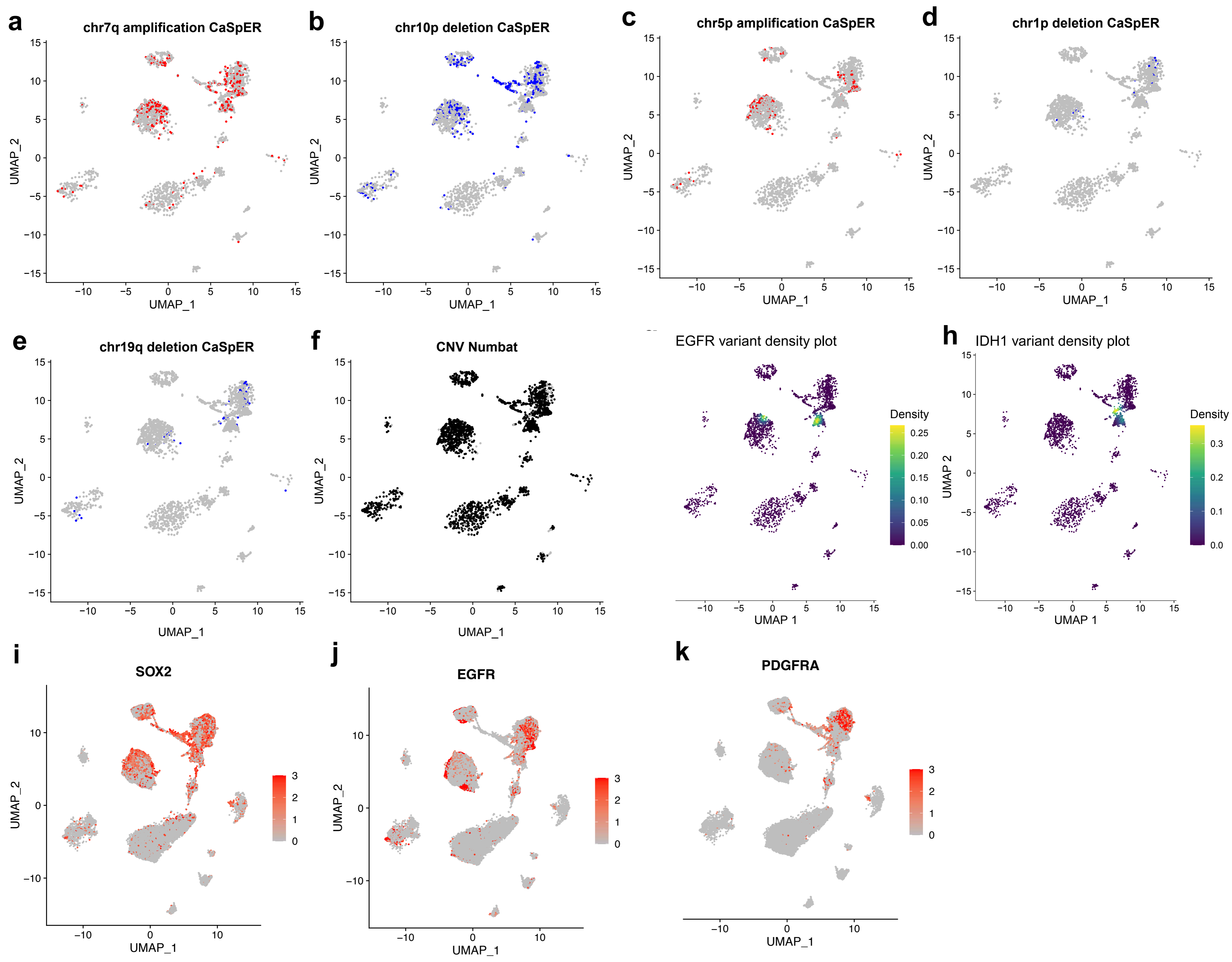

Supplement: Supplementary Figure 1. — Supplementary Figure 1 [file crc-24-0281_supplementary_figure_1.suppsf1.pdf]

# Supp. Figure 2

**a**

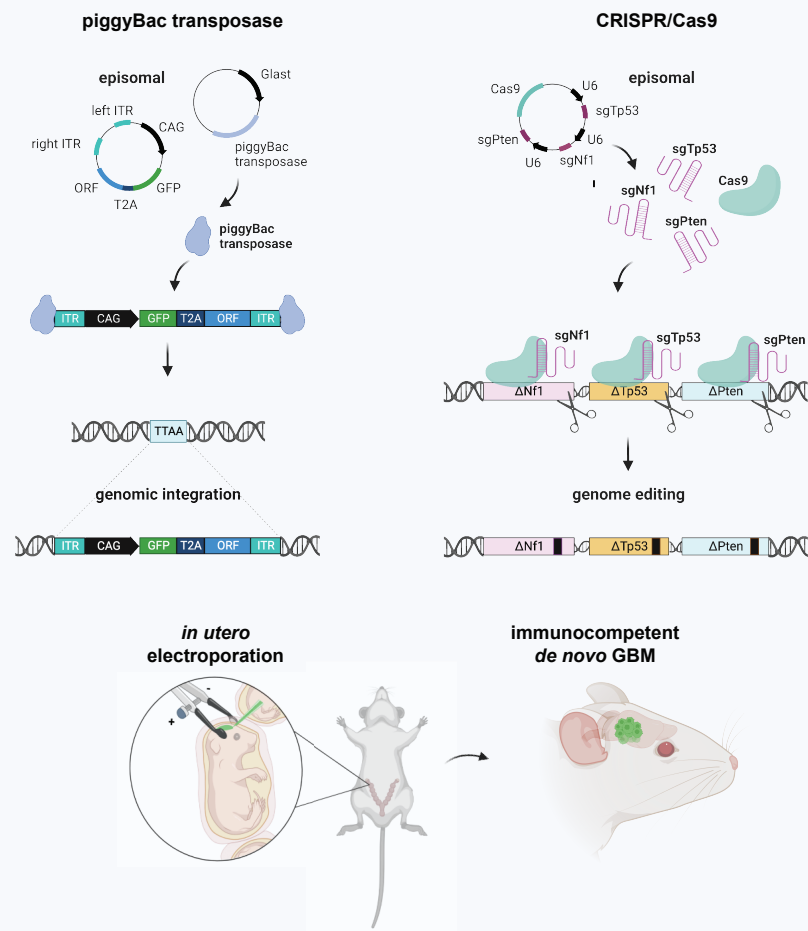

**b**

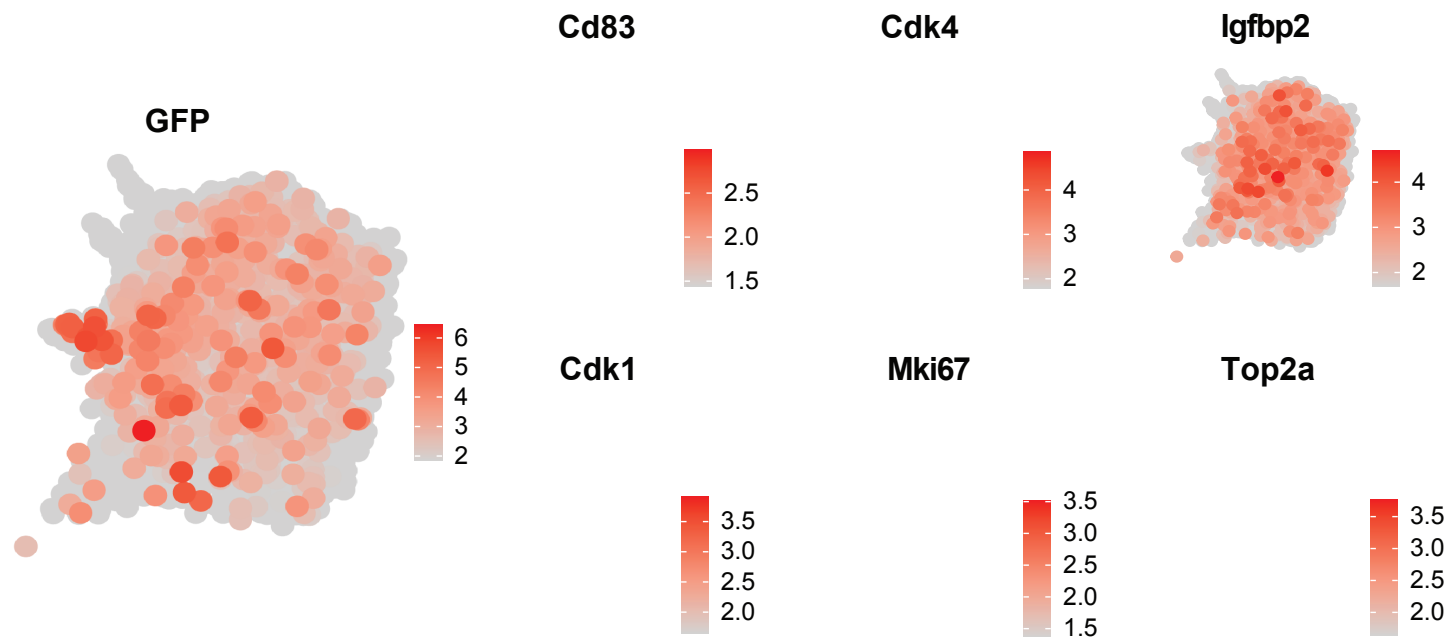

Supplement: Supplementary Figure 2 [file crc-24-0281_supplementary_figure_2_suppsf2.pdf]

# Supp. Figure 3

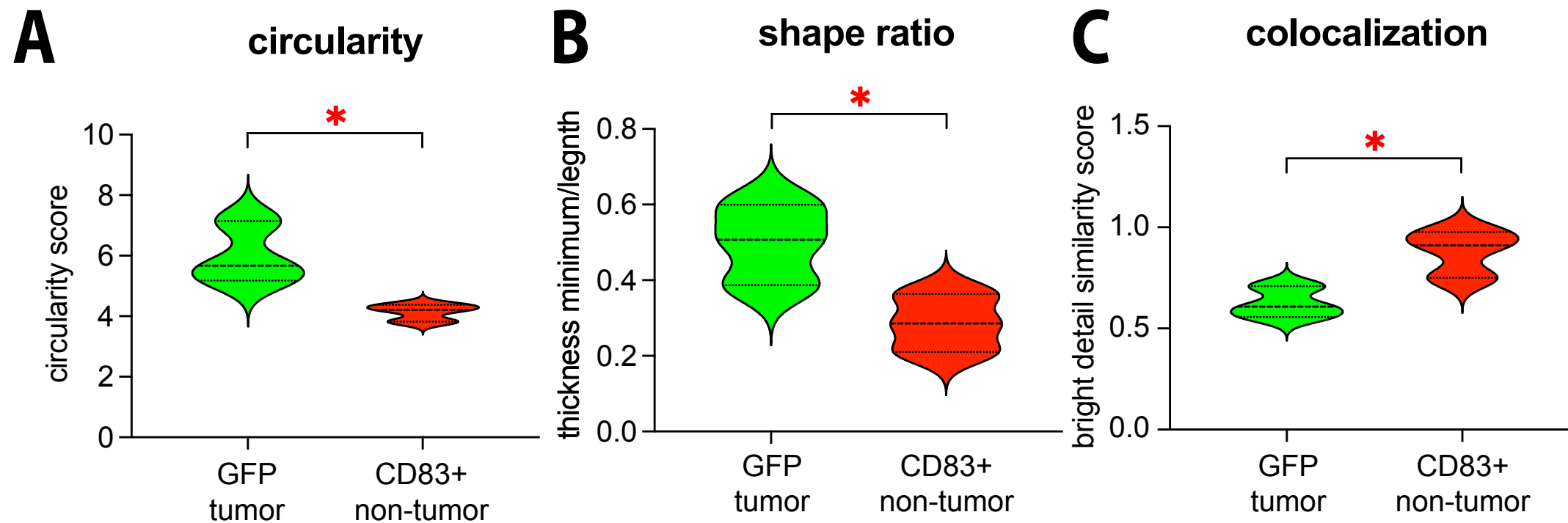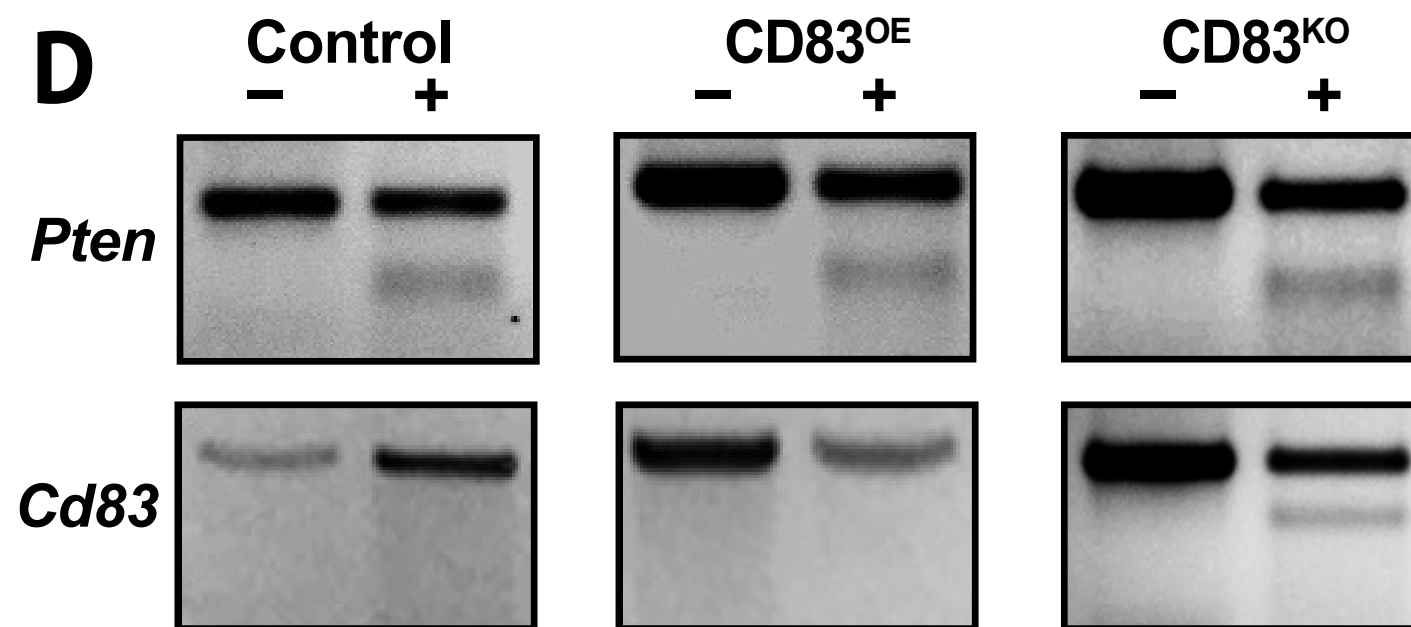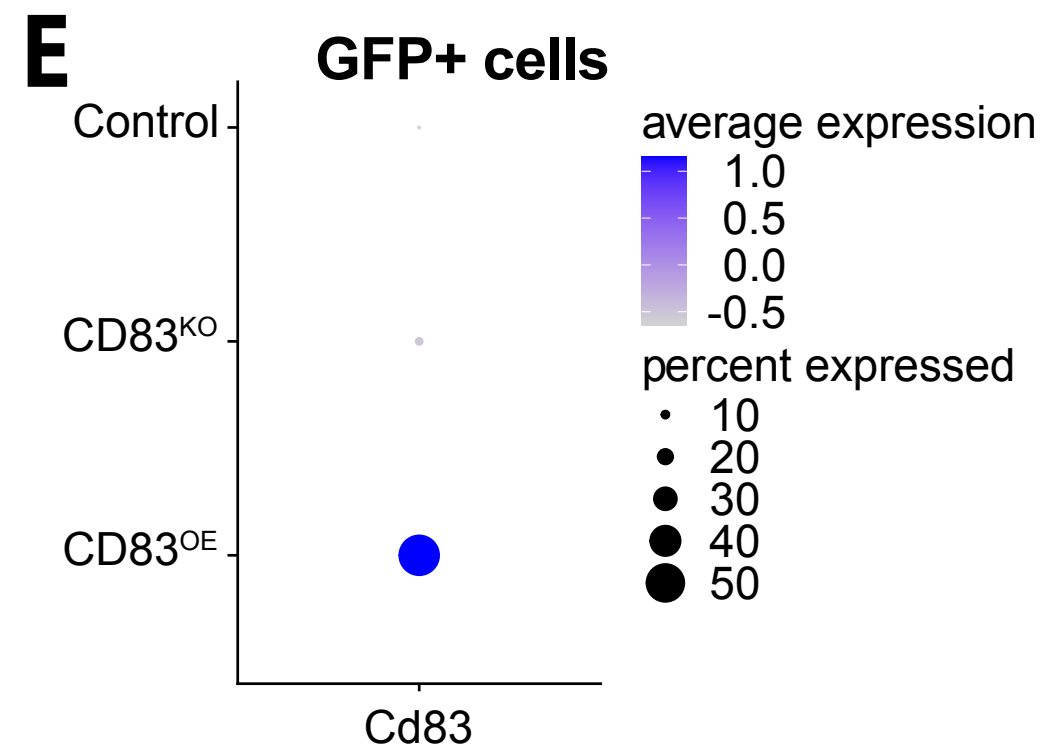

Supplement: Supplementary Figure 3 [file crc-24-0281_supplementary_figure_3.suppsf3.pdf]
