# Supplementary material for: Tumor Expression of CD83 Reduces Glioma Progression and Is Associated with Reduced Immunosuppression
Source: Cancer Res Commun. 2024 Dec 30;4(12):3209–23. doi: 10.1158/2767-9764.CRC-24-0281 (PMC11683667; doi:10.1158/2767-9764.CRC-24-0281)
Supplement: Supplementary Data — Supplemental Figure Legends [file crc-24-0281_supplementary_data_suppsfl.docx]

**Supplementary Figures & Figure Legends**

**Supplementary Figure 1** – **Annotation of tumor cells in scRNAseq**

Seurat plots of CaSpER inferred chromosomal amplifications or deletions for chromosome 7q amplification (a), chromosome 10p deletion (b), chromosome 6p amplification (c), chromosome 1p deletion (d), and chromosome 19q deletion (e) from human IDH^WT^ and IDH^mut^ glioma. Copy number variants inferred using numbat across human scRNAseq (f). Single nucleotide variants for EGFR (g) and IDH1 (h) inferred using XCVATR. Overexpression of common tumor markers for SOX2 (i), EGFR (j), and PDGFRA (l).

**Supplementary Figure 2 – piggyBac *in-utero* electroporation system of murine gliomagenesis recapitulates Cd83+ tumor cells in scRNAseq**

Schematic for design of piggyBac *in-utero* electroporation involving genomic integration and CRISPR mediated deletions of *Nf1*, *Pten*, and *P53* to form immunocompetent, de novo GBM (a). GFP positive tumor cells display expression of Cd83 and other markers of cell-cycle proliferation in scRNAseq (b).

**Supplementary Figure 3 – Characteristics of pB-IUE CD83+ Tumor Cells**

Multispectral flow cytometry characteristics of GFP tumor compared to CD83+ non-tumor showing different distributions of circularity (a), shape ratio (b), and colocalization (c). Detection of insertion/deletion using Surveyor enzyme for *Pten* and *Cd83* (d). Expression of Cd83 in GFP+ tumors cells in scRNAseq across control tumors, CD83^KO^, and CD83^OE^ (e).
